# Supplementary material for: Conditional chemoconnectomics (cCCTomics) as a strategy for efficient and conditional targeting of chemical transmission
Source: eLife. 2024 Apr 30;12:RP91927. doi: 10.7554/eLife.91927 (PMC11060718; doi:10.7554/eLife.91927)
Supplement: Figure 3—source data 3. [file elife-91927-fig3-data3.zip › Figure 3 source data3 part 2.zip!/Figure 3-Source data 3-part2/1062-13-CRISPResso_Report_jt28fhr9.zip!/CRISPResso_on_1062_13/1c.Alignment_barplot.pdf]

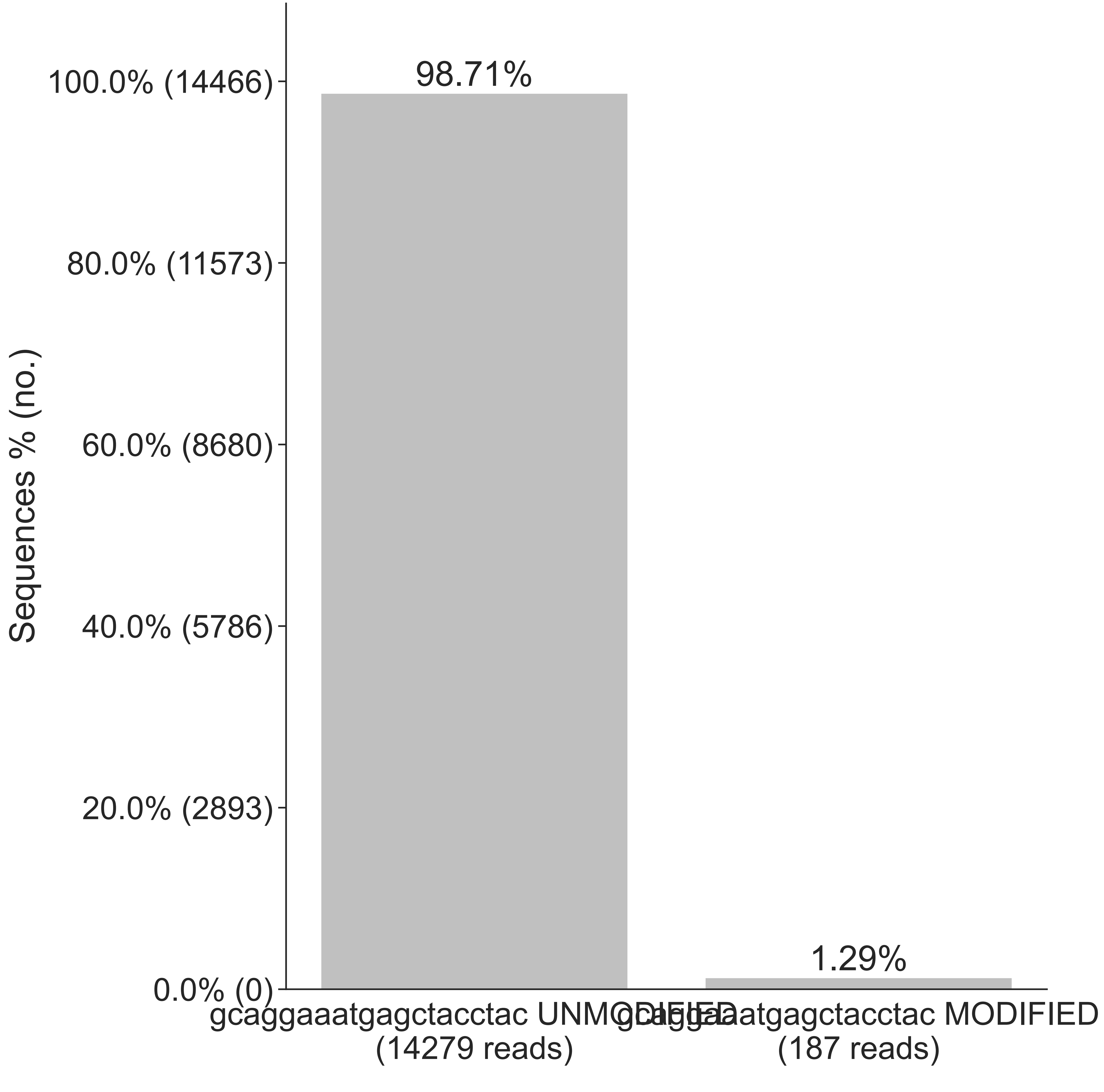

Sequences % (no.)
100.0% (14466)
80.0% (11573)
60.0% (8680)
40.0% (5786)
20.0% (2893)
0.0% (0)
98.71%
1.29%
gcaggaaatgagctacctac UNMODIFIED
(14279 reads)
gcaggaaatgagctacctac MODIFIED
(187 reads)
